# Familial co-aggregation and shared heritability between clinically diagnosed neurodevelopmental problems and cardiometabolic conditions: a nationwide register study across three generations

**DOI:** 10.21203/rs.3.rs-6048644/v1

**Published:** 2025-08-12

**Authors:** Yiran Li, Yiling Zhou, Melissa Vos, Martje Bos, Olivier Steen, Naomi R. Wray, Catharina A. Hartman, Harold Snieder

**Affiliations:** 1 Interdisciplinary Center Psychopathology and Emotion Regulation, Department of Psychiatry, University Medical Center Groningen, University of Groningen, Groningen, Netherlands; 2 Department of Epidemiology, University Medical Center Groningen, University of Groningen, Groningen, Netherlands; 3 Department of Psychiatry and Big Data Institute, University of Oxford, Oxford, United Kingdom; 4 Institute for Molecular Bioscience, The University of Queensland, Brisbane, QLD, Australia

## Abstract

Neurodevelopmental conditions such as attention-deficit/hyperactivity disorder (ADHD) and autism co-occur with cardiometabolic conditions. However, little is known about the mechanisms underlying this co-occurrence. In this nationwide three-generation study using population-based registers in the Netherlands (n=15 million), we assessed the familial (co-)aggregation of ADHD, autism, and cardiometabolic conditions, and estimated their heritabilities and genetic correlations. ADHD, autism, and cardiometabolic conditions showed aggregation and co-aggregation within families and between spouses. Estimated heritabilities of ADHD and autism were moderate (both *h*^2^=0.5), while those of cardiometabolic conditions ranged from low to moderate (*h*^2^=0.1–0.4). Genetic correlations between neurodevelopmental and cardiometabolic conditions were modest (*r*_*g*_ =−0.02–0.20). Together, these results suggest a partly shared familial liability for neurodevelopmental and cardiometabolic conditions, and environmental factors likely play a more important role in the co-occurrence of neurodevelopmental and cardiometabolic conditions than genetics. These new insights can advance research toward specific etiological mechanisms and inform preventive strategies.

Neurodevelopmental conditions are characterized by impairments in cognitive, behavioral, and emotional functioning.^1^ Attention-deficit/hyperactivity disorder (ADHD) and autism are among the most common neurodevelopmental conditions, often manifesting in early childhood and persisting throughout life.^1^ They often co-occur with other psychiatric or somatic conditions, including cardiometabolic conditions,^2,3^ potentially leading to poorer prognostic outcomes.

Epidemiological studies have documented the comorbidity of ADHD and autism with cardiometabolic conditions.^4–8^ A meta-analysis indicated that individuals with ADHD have a more than twofold risk of type-2 diabetes.^4^ Similarly, an increased risk of other cardiometabolic conditions, such as hypertension and obesity, is also present in individuals with ADHD.^5,7^ Although the evidence linking autism to cardiometabolic conditions is less extensive than that on ADHD, a recent meta-analysis confirmed these associations for autism, particularly with diabetes and dyslipidemia.^6^ Recognizing the co-occurrence and understanding mechanisms of the comorbidity can inform on the etiology and aid in developing effective interventions to enhance health outcomes for those affected.

Genetic factors play a substantial role in the development of neurodevelopmental and cardiometabolic conditions. ADHD and autism are highly heritable, with estimated heritabilities ranging from 0.6 to 0.9^9,10^ whereas moderate heritabilities have been shown for various cardiometabolic conditions such as hypertension (0.4), type-2 diabetes (0.5), and heart failure (0.4).^11,12^ However, the extent to which genetic factors contribute to their co-occurrence remains unclear. Recent molecular genetic studies have suggested a potential genetic link between ADHD and cardiometabolic conditions.^13–17^ For example, studies showed that genetic susceptibility to ADHD, represented as a polygenic risk score, is associated with an increased risk of heart failure,^16^ type-2 diabetes, obesity, and hypertension,^17^ while another study used Mendelian randomization indicated that the association between ADHD and type-2 diabetes is likely to be causal.^15^ However, research on why ADHD and especially autism co-occur with cardiometabolic conditions remains scarce.

Progress can be made by analyzing information on ADHD, autism, and cardiometabolic conditions in the context of a family design.^12,18–20^ Using large-scale pedigree data, we can quantify familial co-aggregation between neurodevelopmental and cardiometabolic conditions by estimating the cross-condition risks in relatives. Measures of co-aggregation across various relatives provide insight into the influence of familial factors on the risk of co-occurrence.^21^ For instance, an elevated risk of cardiometabolic conditions in siblings of individuals with ADHD suggests that shared familial factors may contribute to co-occurrence. Family data also allows for the estimation of genetic correlations, which quantifies the extent of genetic sharing between two conditions. Three recent studies using data from Denmark and Sweden have investigated co-aggregation between neurodevelopmental and cardiometabolic conditions within families.^12,18,19^ One of these also estimated genetic correlations.^12^ So far, a modest increased risk of autism was found to be associated with diabetes, obesity, and hypertension in first-degree relatives (1DRs) and second-degree relatives (2DRs).^19^ Similarly, small cross-condition associations in parent-offspring or sibling pairs were found between ADHD, autism, and a broader range of cardiometabolic conditions.^12,18^ Small genetic correlations among parent-offspring pairs of young to middle-age were found between ADHD, autism and cardiometabolic conditions.^12^ Prior studies used only first- and second-degree relatives, which may bias estimates of heritability due to inflation by the shared environment. We attempt to replicate these findings using data on more than 15 million individuals living in the Netherlands and extend these studies by using a larger age range across the lifespan, and including third-degree relatives (3DRs).

In summary, despite the importance of understanding the mechanisms underlying the relationship between neurodevelopmental and cardiometabolic conditions, the knowledge on familial co-aggregation and genetic correlations of these conditions is still sparse. To advance this knowledge, large, genetically informative studies with data on multigenerational relatives across the lifespan are needed. We conducted a large population-based study to investigate the familial (co-)aggregation, heritability, and genetic correlations between neurodevelopmental conditions (i.e., ADHD and autism) and a wide range of cardiometabolic conditions (hypertension, type-2 diabetes, dyslipidemia, stroke, heart failure, angina pectoris, and myocardial infarction) among first-, second-, and third-degree relatives across three generations using national registry data of the entire Dutch population. Using large-scale, population-based registry data, our study is based on a representative sample of the general population, i.e., with a reduced risk of ascertainment biases commonly seen in molecular genetic studies. We additionally validated results of heritability for neurodevelopmental and cardiometabolic conditions using the Dutch Lifelines cohort study, including over 167,000 participants from the northern population of the Netherlands.

## Results

### Descriptive characteristics

As shown in Table 1, we identified 15,266,037 adults with a mean age of 47.8 years (standard deviation, SD: 17.6), among whom 7,872,975 (51.6%) were men. A total of 474,390 (3.1%) individuals had ADHD and 101,625 (0.7%) had autism. The average ages in the ADHD and autism groups were 34.2 (SD: 14.4) and 33.8 (SD: 14.3) years, while they were 48.3 (SD: 17.5) and 47.9 (SD: 17.6) years in the non-ADHD and non-autism group. There were 61.2% and 70.5% males in the ADHD and autism groups, while 51.3% and 51.4% in the non-ADHD and non-autism groups. Prevalences of cardiometabolic conditions in the different groups are shown in Table 1 and ranged from 1.1% for myocardial infarction to 26.3% for hypertension overall. The descriptive characteristics of the Lifelines cohort are shown in **eTable 1**.

### Familial aggregation and heritability

**Figure 1.**
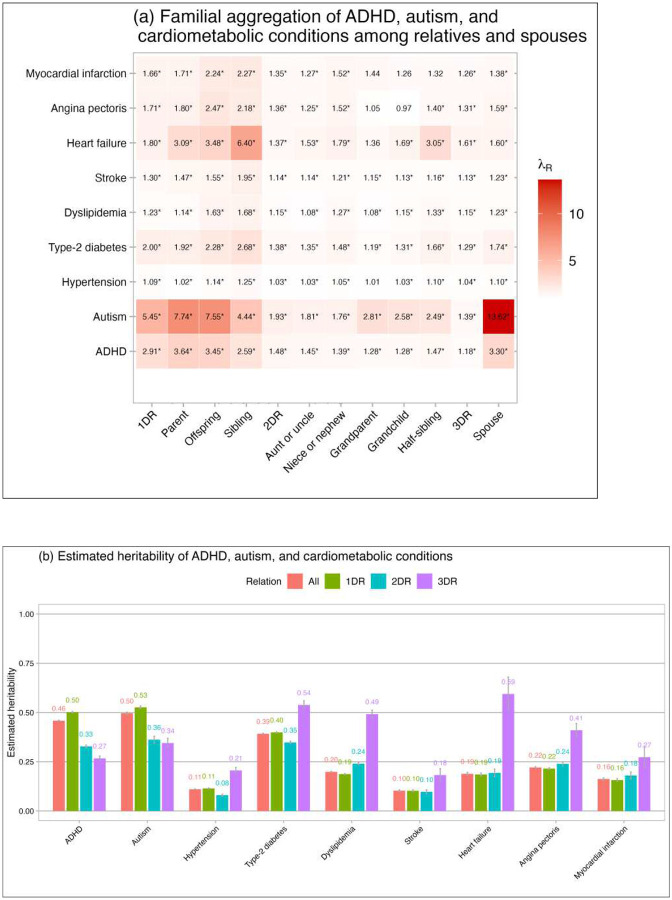
Familial aggregation (a) and estimated heritability with 95% confidence intervals (b) of all conditions Note: ADHD, attention-deficit/hyperactivity disorder; 1DR: (any) first-degree relative; 2DR: (any) second-degree relative; 3DR: (any) third-degree relative (i.e., first cousin). *λ*_*R*_ adjusted for sex, number of the type of relatives, and age (modeled as age and age^2^ to account for non-linear effects) of both individuals and their relatives. The interaction between exposure and age (age and age^2^) and the interaction between exposure and sex were included to account for effect modifiers. *Indicates that the 95% confidence interval does not contain 1.

Positive familial aggregation of neurodevelopmental and cardiometabolic conditions is shown in Figure 1 and **eTable 2**. Generally, having a relative or spouse affected by a certain neurodevelopmental or cardiometabolic condition was associated with a higher risk of the condition compared to the general population. *λ*_*R*_ ranged from 2.91 to 5.45, 1.48 to 1.93, and 1.18 to 1.39 in 1DR, 2DR, and 3DR for neurodevelopmental conditions, respectively, and from 1.09 to 2.00, 1.03 to 1.38, 1.04 to 1.61 in 1DR, 2DR, and 3DR for cardiometabolic conditions, respectively. Increased risks between spouses were also found, with *λ*_*R*_ of 3.30 for ADHD, 13.62 for autism, and a range from 1.10 to 1.74 for cardiometabolic conditions. Based on *λ*_*R*_, heritabilities of neurodevelopmental and cardiometabolic conditions were estimated. Figure 1b shows the overall estimations and the separate estimations from 1DR, 2DR, and 3DR. Overall, ADHD and autism had moderate levels of heritabilities (*h*^2^=0.46 and 0.50, respectively) and cardiometabolic conditions had small-to-moderate levels of heritabilities (*h*^2^ ranged from 0.10 to 0.39). Specifically, the estimates of ADHD and autism from 1DR were higher than 2DR and 3DR, whereas estimates for cardiometabolic conditions were highest in 3DR compared to 2DR and 1DR (based on non-overlap in 95% confidence intervals (CIs)). Results calculated from different relatives are shown in **Supplementary eFigure 1** and **eTable 2**. Heritability estimates from siblings, half-siblings, and first cousins (from the same generation) were comparable (**Supplementary eFigure 1a**). In the sensitivity analyses that including younger age subgroups (18 to 65 and 18 to 55 years), the estimated heritabilities for ADHD and autism were similar to the main results; for cardiometabolic conditions, they were higher than those in the main analyses with increasing estimates in younger age groups (**Supplementary eFigure 2, eTable 3, and eTable 4**). In the Lifelines cohort, heritability estimates for ADHD and autism symptoms (*h*^2^ =0.33 and 0.39, respectively)^22^ were slightly lower than those for clinical ADHD and autism diagnoses from CBS, while estimates for cardiometabolic conditions were in the same small-to-moderate range (*h*^2^ ranged from 0.10 to 0.50) but mostly a bit higher than those from CBS (**Supplementary eFigure 3**).

### Familial co-aggregation and genetic correlations

**Figure 2.**
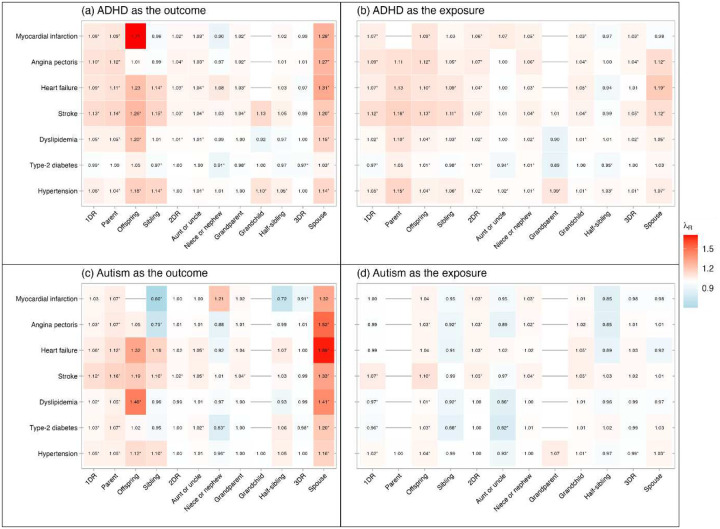
Familial co-aggregation of ADHD, autism with cardiometabolic conditions Note: ADHD, attention-deficit/hyperactivity disorder; 1DR: (any) first-degree relative; 2DR: (any) second-degree relative; 3DR: (any) third-degree relative (i.e., first cousin). Figures (a) and (c) showed the *λ*_*R*_ of ADHD or autism in individuals with relatives affected by cardiometabolic conditions; figures (b) and (d) showed the *λ*_*R*_ of cardiometabolic conditions in individuals with relatives affected by ADHD or autism. *λ*_*R*_ adjusted for sex, number of the type of relatives, and age (modeled as age and age^2^ to account for non-linear effects) of both individuals and their relatives. The interaction between exposure and age (age and age^2^) and the interaction between exposure and sex were included to account for effect modifiers. —: Results were unavailable due to the limited number of cases for certain exposure-outcome combinations (e.g., when parental autism was modeled as the exposure and cardiometabolic conditions in the offspring as the outcome). *Indicates that the 95% CI does not include 1.

The results of familial co-aggregation analyses of ADHD and autism with cardiometabolic conditions are represented in Figure 2 and **eTable 5**. There was positive co-aggregation between ADHD and cardiometabolic conditions in 1DR (*λ*_*R*_ ranged from 1.02 to 1.13), except for type-2 diabetes (*λ*_*R*_=0.99 and 0.97, Figure 2a, 2b). In 2DR and 3DR, co-aggregation was found for most cardiometabolic conditions, but the effects were smaller (*λ*_*R*_ ranged from 0.97 to 1.07). Spouses also showed positive co-aggregation for most cardiometabolic conditions (*λ*_*R*_ ranged from 0.98 to 1.31). For autism, positive co-aggregation was found in most scenarios when autism was modelled as the outcome and cardiometabolic conditions of ancestors were modelled as the exposure, showing an increased risk of autism in individuals with ancestors affected by cardiometabolic conditions (parent, aunt or uncle, and grandparent in Figure 2c, *λ*_*R*_ ranged from 1.00 to 1.16). Similarly, positive co-aggregation was found in most scenarios when cardiometabolic conditions were modelled as the outcome and autism of descendants was modelled as the exposure (i.e., offspring, niece or nephew, and grandchild in Figure 2d, *λ*_*R*_ ranged from 1.00 to 1.10). Among spouses, positive co-aggregation was shown when autism was modelled as the outcome, but not as the exposure.

**Figure 3.**
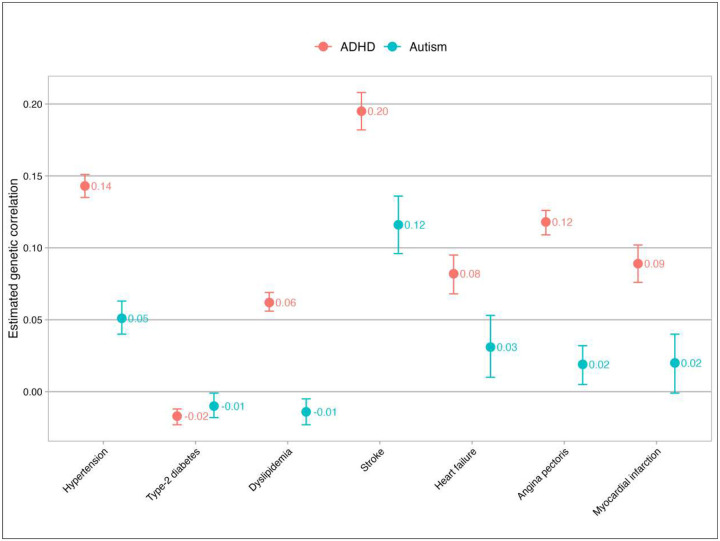
Estimated genetic correlations with 95% confidence intervals between ADHD, autism and cardiometabolic conditions Note: ADHD, attention-deficit/hyperactivity disorder.

Based on these cross-condition *λ*_*R*_ values, small positive genetic correlations between ADHD and cardiometabolic conditions were found, ranging from 0.06 to 0.20 except for type-2 diabetes (r_g_=−0.02, Figure 3). For autism, small positive genetic correlations with hypertension, stroke, heart failure, and angina pectoris were found (r_g_ ranged from 0.02 and 0.12) and small negative genetic correlations with type-2 diabetes and dyslipidemia was found (r_g_=−0.01, Figure 3). A substantial genetic correlation of 0.56 (95% CI: 0.55–0.57) was found between ADHD and autism. In the sensitivity analyses that including younger age subgroups (18 to 65, and 18 to 55 years), the estimated genetic correlations were similar to those of the main results (**Supplementary eFigure 4, eTable 6**, and **eTable 7**). Environmental factors played a comparable or larger role than genetic factors in explaining the comorbidity (**Supplementary eFigure 5**). The Odds Ratios (ORs) used to calculate phenotypic correlations between each pair of conditions were shown in **eTable 8**.

## Discussion

Including over 15 million samples across 3 generations, this is one of the largest and comprehensive studies to estimate the familial (co-)aggregation, heritabilities, and genetic correlations of neurodevelopmental and cardiometabolic conditions. Individuals who had a family member with one of these conditions (e.g., ADHD) were at a higher risk of developing the same (ADHD), another neurodevelopmental (autism) or other, cardiometabolic conditions (e.g., hypertension), reflecting the co-occurrence of neurodevelopmental and cardiometabolic conditions within families. These patterns were similar for autism. Similar increased risks were also found in individuals with an affected spouse, indicating an effect of shared environmental factors or assortative mating. The estimated heritabilities of all conditions were low to moderate, and the estimated genetic correlations between neurodevelopmental and cardiometabolic conditions were modest. The modelling approach inferred that the comorbidity of these cardiometabolic traits with ADHD or autism originated more from environmental than genetic factors. These findings indicate that genetics play some, though certainly not exclusive, role in the associations of neurodevelopmental with cardiometabolic conditions.

Our results of positive co-aggregation among relatives support the findings of recent studies that mostly included limited subsets of 1DRs (e.g., parent-offspring, sibling pairs),^12,18^ and extend findings to 2DRs and 3DRs. In accordance with expectations, recurrence risk ratios estimated in 2DRs and 3DRs were lower compared to those in 1DRs, reflecting the effect of reduced genetic relatedness and shared environmental factors. For ancestors, more positive co-aggregation was observed when ADHD or autism was the outcome (i.e., when ancestors had cardiometabolic conditions), compared to when ADHD or autism was the exposure (i.e., when ancestors had ADHD or autism). For descendants we found the opposite, i.e., more positive co-aggregation was observed when ADHD or autism was the exposure (i.e., when descendants had ADHD or autism), compared to when ADHD or autism was the outcome (i.e., when descendants had cardiometabolic conditions). These differences were also expected, given that cardiometabolic conditions typically have an adulthood onset and neurodevelopmental conditions were often underdiagnosed in older generations. Apart from these patterns of familial co-aggregation, modest genetic correlations of ADHD and autism with cardiometabolic conditions were found, which further indicates that the observed comorbidity can be mostly attributed to environmental factors. Our estimations of genetic correlations generally align with those reported in a previous family study using Scandinavian registry data^12^ (**Supplementary eFigure 6**), with the exception that we did not observe a genetic correlation between ADHD and type 2 diabetes. Despite the role of genetics, our results highlight the greater importance of environmental factors, which constitute a major component of the comorbidity, underscoring the need for more studies focused on environmental influences underlying these comorbidities.

Corresponding with previous evidence,^23,24^ we found that spouses of individuals with ADHD and autism also had substantially higher risks of having these conditions compared to the general population. Since ADHD and autism typically have an early onset, the increased risks are likely attributable to assortative mating (e.g., partner selection based on similarities in the phenotype). Regarding co-aggregation, the increased risk of cardiometabolic conditions among spouses of individuals with ADHD or autism may also reflect shared environmental factors within families besides assortative mating. It is well established that shared lifestyle factors, such as alcohol consumption, smoking, and physical activity, contribute to the co-occurrence of cardiometabolic conditions among spouses.^25,26^

Our study showed that the estimated heritabilities of both ADHD and autism were moderate, with estimates of approximately 0.5, while the heritability of cardiometabolic conditions were small to moderate, ranging between 0.1 to 0.4. Compared to a previous family study using Scandinavian registry data,^12^ our heritability estimates are generally comparable, with the exception of heart failure, for which we observed a higher estimate (**Supplementary eFigure 7**). Compared to those typically reported in twin studies,^27–29^ our estimates are generally lower. Twin studies have reported the heritability of ADHD and autism to be between 0.6–0.9;^27,28^ and a recent meta-analysis based on twin studies reported the heritabilities of cardiovascular and metabolic conditions in the range of 0.4–0.6.^29^ The variability in heritability estimates with twin studies can have multiple reasons. First, diagnostic criteria are more homogeneous within the same generations (i.e., twins have the same age) than in relatives across generations. For example, neurodevelopmental conditions were historically under- or mis-diagnosed in older generations due to different diagnostic criteria and limited public awareness,^30^ leading to greater discordance in relatives across generations than those within the same generation. Second, genetic influence may vary with age, resulting in lower concordance among family members across generations than within the same generation.^31,32^ Third, the effect of right censoring (i.e., not all participants have reached a given age to be affected by cardiometabolic conditions) is more pronounced within relatives across generations. These factors are evident in our results. For cardiometabolic conditions, heritability estimates from 3DR (i.e., first cousins, usually from the same generation) were higher compared to 1DR and 2DR (Figure 1b) but comparable with those from siblings and half-siblings (**Supplementary eFigure 1a**). For ADHD and autism, the heritability estimates from 1DR were higher than 2DR and 3DR (Figure 1b), which may be attributed to the increased likelihood of identifying neurodevelopmental conditions within immediate family relationships. That is, a diagnosis of ADHD or autism in a child may increase recognition of the symptoms in the parent who may then receive a late diagnosis, or vice versa: parents with the diagnosis may be aware of possible intergenerational transmission lowering the threshold to consult a clinician —an effect less pronounced in 2DRs (e.g., aunt and niece) and 3DRs. Thus, the variations in heritability estimates across generations within our study and compared to twin studies likely reflect factors such as diagnostic bias, effects of gene-age interaction, and right-censoring.

In the sensitivity analyses examining younger age subgroups (18 to 55 and 18 to 65 years), the heritability estimates for ADHD and autism were similar to those of the main results. For cardiometabolic conditions, the higher estimates suggest stronger genetic influences on early-onset forms of these conditions. Comparing with the results from CBS, heritability estimates for ADHD and autism symptoms in Lifelines were slightly lower,^22^ which could be attributed to the differences between measurements of clinical diagnoses in CBS and self-report questionnaires in Lifelines. The presence of a clinical diagnoses depends on help seeking and therefore are focused on only severe (clinical) problems while the questionnaires were continuous measures of symptom severity which are more sensitive to less severe symptoms. Regarding cardiometabolic conditions, we found that Lifelines showed somewhat higher heritability estimates (0.1–0.5, **Supplementary eFigure 3**) compared to our main results (0.1–0.4, Figure 1b). The observed variations may be attributed to disparities between the more severe clinical diagnoses in CBS compared with those based on the likely more sensitive measures of self-reported diagnoses in combination with medication use and/or objective biomarkers (e.g., blood pressure for hypertension and HBA1c and fasting glucose for type-2 diabetes, lipids for dylipidemia, electrocardiograms for myocardial infarction) in Lifelines.

Overall, our study contributes to the literature by providing comprehensive, genetically informative parameter estimates of neurodevelopmental and cardiometabolic conditions. Our results suggested that etiological mechanisms of the co-occurrence between neurodevelopmental and cardiometabolic conditions originated more from environmental than genetic factors. These environmental factors could be multifactorial and related to lifestyle factors such as physical activity, smoking, and sleeping problems.^33–35^ Previous studies have shown positive effects of physical exercise on cardiometabolic health and autistic symptoms in individuals with autism.^36–38^ Therefore, to reduce the risk of cardiometabolic conditions, lifestyle interventions, such as physical activity promotion and tobacco use prevention may be particularly beneficial for individuals with neurodevelopmental conditions.^39^

### Limitations

Several study limitations need to be noted. First, the methods used to estimate heritability are based on modelling assumptions. In calculation of risk ratios, it is assumed that the observed risks in the general population and each relative class represent the lifetime risk of the condition (or risk to a given age); even in perfect recording of data, there will be right censoring as not all participants have reached a given age. Age and age-squared are used as covariates which mitigates this to some degree. Second, the method does not explicitly model shared environmental effects between family members. The validity of this assumption can be explored through the comparison of heritability estimates made from different types of relatives. In the absence of shared environmental factors, estimates should be consistent. For example, the heritability estimated from siblings, half-siblings, and cousins were comparable, suggesting that the bias due to shared environment is minimal. Third, the method used to estimate heritability assumes random mating, whereas high spouse correlations for some traits (particularly ADHD and autism) implies assortative mating. The presence of assortative mating can (modestly) increase estimates of heritability^23^ and genetic correlations.^40^ Fourth, our study relied on the national registry data CBS and the ascertainment of all conditions was based on CBS data during the available period (i.e., diagnoses recorded and made by specialists between 2013 and 2020 and medication dispensation from both specialists and general practitioners (GPs) between 2006 and 2020). Our results are influenced by left censoring, meaning that diagnoses made before the available data period may not have been fully captured. For ADHD and autism, our data will likely have captured most severe cases as these conditions are lifelong conditions and typically diagnosed by specialists in the Netherlands. However, cases identified solely in general practice, without subsequent referral or pharmacological treatment (in the case of ADHD) may have been missed. This may lead to misclassification of milder cases, although such cases are likely to be rare. Similarly, for cardiometabolic conditions, we may also have missed classification of some milder cases. Yet, our strategy of integrating medication dispensations from 2006 to 2020 to identify ADHD and the less severe cases of cardiometabolic conditions (including hypertension, type-2 diabetes, and dyslipidemia), partially controlled for these biases. Fifth, although the medications we used to ascertain ADHD are authorised for the treatment of ADHD, off-label use is possible. For example, methylphenidate has occasionally been prescribed during the terminal stages of cancer, especially among individuals over 50 years.^41^ Nonetheless, we expect the number of such cases to be very small and unlikely to affect our findings. Finally, conduct disorder, oppositional defiant disorder, and other behavioral disorders were also identified as ADHD and Rett’s disorder as autism from 2017 to 2020 due to the registry coding system not distinguishing these conditions starting in 2017. However, based on data from 2013 to 2016—when these conditions could be identified—we estimated that such cases accounted for approximately 7% of the total ADHD group and 4% of the total autism group. Assuming a similar distribution during the 2017–2020 period—which accounts for 50% of the total study duration—the estimated false-positive cases represent approximately 3.5% (7% × 0.5) and 2% (4% × 0.5) of all ADHD and autism cases, respectively, across the entire study period 2016–2020. Although these misclassification rates will have reduced the precision of our results somewhat, their impact will likely have been small.

## Conclusions

In conclusion, ADHD, autism and cardiometabolic conditions showed aggregation and co-aggregation within families and between spouses. We estimated the heritabilities for ADHD and autism as moderate, and small to moderate for cardiometabolic conditions. Further, genetic correlations between ADHD, autism and cardiometabolic conditions were modest, and the observed comorbidities could mostly be attributed to environmental factors. Our genetically informative design allowed us to examine the familial liability, and highlighted the influence of environmental factors more than genetic factors underlying etiological mechanisms. Future population-based and molecular genetic studies are needed to uncover specific mechanisms in the cross-condition patterns within families.

## Methods

### Data source

In this population-based study, we linked multiple Dutch nationwide registers derived from Statistics Netherlands (Centraal Bureau voor de Statistiek, CBS).^42^ The Municipal Personal Records Database (GBA, since 1994) consists of the demographic data of the entire Dutch population. It was used to identify the study population and obtain information on the birth date, sex, emigration status, and date of death. The Family Network Register (since 1994), which includes all family relationships (e.g., nuclear, multi-generational, and spousal) of the Dutch population, was used to identify kinships. The Medical Claim Register (since 2013) includes somatic and psychiatric conditions diagnosed by specialists (outpatient and inpatient). The Pharmaceutical Care Claim Register (since 2006)^43^ included all medications prescribed in primary and specialist care, coded based on the Anatomical Therapeutic Chemical (ATC) Classification System.

We additionally validated results of heritability for neurodevelopmental and cardiometabolic conditions using the Dutch multigenerational Lifelines cohort study.^44^ A detailed description is provided in the Supplementary materials. Briefly, Lifelines is a population-based cohort from the north of the Netherlands, which includes self-reported symptom measures for ADHD and autism, as well as cardiometabolic conditions based on biomarkers, self-report of diagnoses and medication use. This allowed us to assess the consistency of our registry-based findings in a cohort study using different measurements.

### Study Population

We defined the general population in our study as all adults born between 1940 and 2002 (aged 18–80 years in 2020 at the end of the study period) and excluded those who had emigrated before December 31, 2020 or died before January 1, 2013. Using the Dutch Family Network Register, we identified 1DRs (including parents, full siblings, and offspring), 2DRs (including grandparents, grandchildren, aunts or uncles, nieces or nephews, and half-siblings), 3DRs (i.e., first cousins), and spouses among these individuals. The sample sizes of each type of family relationships are presented in **eTable 2**. The use of Dutch register data does not require informed consent.

### Ascertainment of ADHD, autism and cardiometabolic conditions

All conditions were ascertained using available medical claims data (diagnoses between 2013 to 2020 and medication dispensation between 2006 to 2020). Individuals with ADHD were identified as those who had either received an ADHD diagnosis based on Diagnostic and Statistical Manual of Mental Diseases, 4th edition (DSM-IV) (**eTable 9**, i.e., ADHD between 2013 and 2016, attention-deficit and disruptive behavior disorders between 2017 and 2020 (due to changes in coding in the register, including ADHD, as well as conduct disorder, oppositional defiant disorder, and behavioral disorder) or an ADHD medication prescription (ATC codes: N06BA01, N06BA02, N06BA04, N06BA09, and N06BA12).^45^ Conduct disorder, oppositional defiant disorder, and behavioral disorder without ADHD together comprised 3.5% of the total. Individuals with autism were identified as those who had received an autism diagnosis based on the DSM-IV (**eTable 9**), including autistic disorder, Rett’s disorder, childhood disintegrative disorder, Asperger syndrome, and pervasive developmental disorders-not otherwise specified. Rett’s disorder constituted 2% of the total.

We identified individuals with cardiometabolic conditions (including hypertension, type-2 diabetes, dyslipidemia, stroke, heart failure, angina pectoris, and myocardial infarction) as those who received the corresponding diagnosis codes listed in **eTable 9**. For hypertension (ATC codes: C02, C03, C07, C08, and C09), type-2 diabetes (ATC code: A10B), and dyslipidemia (ATC code: C10), we also identified those that received a medication prescription.

### Statistical Analysis

#### Familial (co-)aggregation

To examine the familial (co-)aggregation, recurrence risk ratios (*λ*_*R*_) were estimated. *λ*_*R*_ is the ratio of the prevalence of the condition in relatives of affected participants (e.g., siblings) compared to the prevalence in the general population.^46^ We employed plugin methods to estimate the marginal *λ*_*R*_ over covariates. First, we fitted logistic regression models regressing the outcome on the exposure (e.g., whether a sibling was affected by the condition). Models were adjusted for sex, number of relatives, and age (modelled as age and age^2^ to account for non-linear effects) of both the individual and the relative. The interaction between exposure and age (age and age^2^) and the interaction between exposure and sex were included to account for effect modifiers.

Second, we estimated the average prevalence of the condition among the total population by plugging-in the fitted logistic regression to the observed total population; and the marginal prevalence of the condition among individuals with an affected sibling, calculated by plugging-in the fitted logistic regression to the observed total population data, with the exposure set to 1 for all observations.^47,48^ To account for familial clustering and the resulting within-family correlations, 95% confidence intervals (CIs) were estimated for each prevalence using a robust sandwich estimator.^49^ Standard errors (SE) of *λ*_*R*_ were estimated using the delta method.^50^ *λ*_*R*_ was also calculated for other relatives. These analyses were conducted using the R package “marginaleffects”.^51,52^ See Supplementary methods for details.

#### Heritability (h^2^) and genetic correlation (r_g_)

While *λ*_*R*_ are directly estimated from observed data, they can be hard to compare across conditions because of the differences in base-rate prevalences. Comparison of familial aggregation can be benchmarked using genetic parameter estimation, which is conducted under model assumptions. Hence, we estimated the heritability (*h*^2^) and genetic correlation (*r*_*g*_) based on the liability threshold model. This approach, originally developed by Falconer^53^ and Reich et al.^54^ and later adapted by Wray and Gottesman,^55,56^ has been previously applied to register data with dichotomous outcomes.^55^ A detailed description can be found in the Supplementary methods. Briefly, the liability threshold model assumes that the liability of the condition underlying the condition status is normally distributed and that individuals with the condition must have surpassed a liability threshold.^53,55,56^ Based on the liability threshold model, the heritability and genetic correlation can be estimated using *λ*_*R*_, prevalence of the condition in the total population, and the genetic relatedness between relatives (0.5 for 1DRs, 0.25 for 2DRs, and 0.125 for 3DRs). The simple modelling approach assumes that the observed increased risk in relatives is attributed only to genetic factors. Heritabilities were estimated for different relative pairs, so that consistency of estimates can be considered with respect to underlying assumptions. The overall *h*^2^ and *r*_*g*_ were estimated using a random-effects inverse variance weighted model, weighting the estimations of any 1DR, any 2DR, and any 3DR by the inverse of their sampling variance.

#### Contributions of genetic and environmental factors

Under a bivariate liability threshold model, the phenotypic covariances between ADHD, autism, and cardiometabolic conditions could be partitioned into genetic and environmental factors, which allowed us to quantify the relative contributions of the genetic and environmental factors to the observed comorbidity.^12,57^ Phenotypic covariance was calculated from phenotypic correlations, which were estimated using Odds Ratio (OR) between each pair of conditions (i.e., associations between two conditions within individuals adjusted for age and sex).^58,59^ The environmental factor was calculated using a mathematical formula that included the estimates of phenotypic correlation, heritability, and genetic correlation. See Supplementary methods for details.

#### Sensitivity analyses

Considering the historical underdiagnosis of ADHD and autism among older individuals, we conducted age-stratified sensitivity analyses in two subgroups with narrower age ranges: (1) individuals aged 18–65 years (i.e., born between 1955 and 2002), and (2) individuals aged 18–55 years (i.e., born between 1965 and 2002).

## Supplementary Material

Supplementary Files

This is a list of supplementary files associated with this preprint. Click to download.

- SupplementMaterials.pdf
- SupplementTables.pdf

## Figures and Tables

**Table 1.** Descriptive characteristics of the samples

Characteristics	Overall	ADHD	Non-ADHD	Autism	Non-autism
N (%)	15,266,037 (100)	474,390 (3.1)	14,791,647 (96.9)	101,625 (0.7)	15,164,412 (99.3)
Age, year, mean (SD)	47.8 (17.6)	34.2 (14.4)	48.3 (17.5)	33.8 (14.3)	47.9 (17.6)
Birth year, n (%)					
[1940, 1950)	1,961,391 (12.8)	9,506 (2.0)	1,951,885 (13.2)	1,422 (1.4)	1,959,969 (12.9)
[1950, 1960)	2,351,685 (15.4)	20,965 (4.4)	2,330,720 (15.8)	4,944 (4.9)	2,346,741 (15.5)
[1960, 1970)	2,701,404 (17.7)	46,245 (9.7)	2,655,159 (18.0)	10,417 (10.3)	2,690,987 (17.7)
[1970, 1980)	2,389,613 (15.7)	61,960 (13.1)	2,327,653 (15.7)	12,712 (12.5)	2,376,901 (15.7)
[1980, 1990)	2,518,605 (16.5)	81,404 (17.2)	2,437,201 (16.5)	16,422 (16.2)	2,502,183 (16.5)
[1990, 2002]	3,343,339 (21.9)	254,310 (53.6)	3,089,029 (20.9)	55,708 (54.8)	3,287,631 (21.7)
Male, n (%)	7,872,975 (51.6)	290,107 (61.2)	7,582,868 (51.3)	71,661 (70.5)	7,801,314 (51.4)
Hypertension, n (%)	4,014,427 (26.3)	99,978 (21.1)	3,914,449 (26.5)	19,222 (18.9)	3,995,205 (26.3)
Type-2 diabetes, n (%)	902,487 (5.9)	15,994 (3.4)	886,493 (6.0)	3,696 (3.6)	898,791 (5.9)
Dyslipidemia, n (%)	2,361,668 (15.5)	32,989 (7.0)	2,328,679 (15.7)	6,684 (6.6)	2,354,984 (15.5)
Stroke, n (%)	405,318 (2.7)	7,747 (1.6)	397,571 (2.7)	1,385 (1.4)	403,933 (2.7)
Heart failure, n (%)	176,274 (1.2)	2,316 (0.5)	173,958 (1.2)	440 (0.4)	175,834 (1.2)
Angina pectoris, n (%)	389,909 (2.6)	5,674 (1.2)	384,235 (2.6)	938 (0.9)	388,971 (2.6)
Myocardial infarction, n (%)	169,704 (1.1)	2,267 (0.5)	167,437 (1.1)	423 (0.4)	169,281 (1.1)

Note: ADHD, attention-deficit/hyperactivity disorder; SD, standard deviation.

## Data Availability

Under certain conditions, microdata from CBS are accessible for statistical and scientific research. For further information: microdata@cbs.nl. Data of Lifelines may be obtained from a third party and are not publicly available. Researchers can apply to use the Lifelines data used in this study. More information about how to request Lifelines data and the conditions of use can be found on their website (https://www.lifelines-biobank.com/researchers/working-with-us)

## Code availability

Code for the main analysis is publicly available at https://github.com/yiranli-hi/Familial-coaggregation-project
